# Simplified methods for variance estimation in microbiome abundance count data analysis

**DOI:** 10.3389/fgene.2024.1458851

**Published:** 2024-10-21

**Authors:** Yiming Shi, Lili Liu, Jun Chen, Kristine M. Wylie, Todd N. Wylie, Molly J. Stout, Chan Wang, Haixiang Zhang, Ya-Chen T. Shih, Xiaoyi Xu, Ai Zhang, Sung Hee Park, Hongmei Jiang, Lei Liu

**Affiliations:** ^1^ Institute for Informatics Data Science and Biostatistics, Washington University in St. Louis, St. Louis, MO, United States; ^2^ Division of Computational Biology, Department of Quantitative Health Sciences, Mayo Clinic, Rochester, MN, United States; ^3^ Department of Pediatrics, Washington University, St. Louis, MO, United States; ^4^ Department of Obstetrics and Gynecology, University of Michigan School of Medicine, Ann Arbor, MI, United States; ^5^ Department of Population Health, Division of Biostatistics, New York University Grossman School of Medicine, New York, NY, United States; ^6^ Center for Applied Mathematics, Tianjin University, Tianjin, China; ^7^ Department of Radiation Oncology, Department of Health Policy and Management, School of Medicine, School of Public Health, University of California Los Angeles Jonsson Comprehensive Cancer Center, Los Angeles, CA, United States; ^8^ Department of Pathology and Immunology, Division of Laboratory and Genomic Medicine, and the Edison Family Center for Genome Sciences and Systems Biology, Washington University School of Medicine, St. Louis, MO, United States; ^9^ Department of Statistics and Data Science, Northwestern University, Evanston, IL, United States

**Keywords:** microbiome abundance count, robust variance estimation, heteroscedasticity, sandwich estimates, bootstrap

## Abstract

The complex nature of microbiome data has made the differential abundance analysis challenging. Microbiome abundance counts are often skewed to the right and heteroscedastic (also known as overdispersion), potentially leading to incorrect inferences if not properly addressed. In this paper, we propose a simple yet effective framework to tackle the challenges by integrating Poisson (log-linear) regression with standard error estimation through the Bootstrap method and Sandwich robust estimation. Such standard error estimates are accurate and yield satisfactory inference even if the distributional assumption or the variance structure is incorrect. Our approach is validated through extensive simulation studies, demonstrating its effectiveness in addressing overdispersion and improving inference accuracy. Additionally, we apply our approach to two real datasets collected from the human gut and vagina, respectively, demonstrating the wide applicability of our methods. The results highlight the efficacy of our covariance estimators in addressing the challenges of microbiome data analysis. The corresponding software implementation is publicly available at https://github.com/yimshi/robustestimates.

## 1 Introduction

Human microbiome research has significantly advanced our understanding of microbial communities within the human body and their extensive impacts on human health. The high-throughput sequencing technologies have enhanced our ability to collect and analyze vast amounts of sequencing data.

One critical aspect of microbiome research is differential abundance analysis, which aims to identify microbial taxa whose abundance levels differ significantly between different groups or conditions. This analysis is crucial for understanding how microbial communities are associated with various health conditions, environmental factors, or other biological states (Paulson et al., 2013; Turnbaugh et al., 2007; Qin et al., 2010). By revealing these associations, researchers can gain insights into the complex relations between the microbiome and host, potentially leading to new therapeutic strategies.

In differential abundance analysis, modelling microbiome count data presents unique challenges, particularly due to complex nature in microbiome datasets such as right skewness, heteroscedasticity, and excess zeros. In this study we focused on finding a simple yet effective approach to address heteroscedasticity. To further illustrate heteroscedasticity in microbiome data, we use the genus *Streptococcus* in the adenomas microbiome data (introduced in the simulation section) as an example. *Streptococcus* is a clinically important genus commonly found in the human microbiome. It includes both commensal species that are part of the normal microbiota and pathogenic species associated with infections such as streptococcal pharyngitis and pneumonia. Due to its varying abundance across different individuals and conditions, *Streptococcus* is an ideal candidate for demonstrating the variability in microbiome data.

**FIGURE 1:**
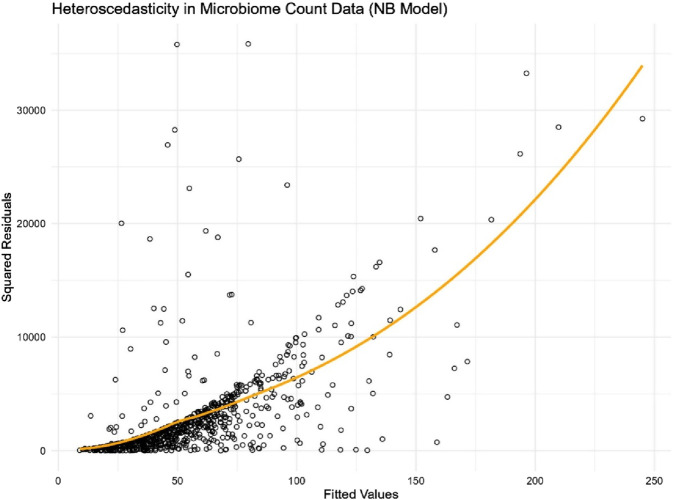
Scatter plot of squared residuals versus fitted values for genus *Streptococcus* in the adenomas microbiome data.

We fitted a Negative Binomial regression model using the microbiome abundance count of this genus against the covariates of interest. Presented in Figure 1, the plot of squared residuals versus fitted values clearly shows an increasing trend in variance as the count values rise. This trend is further validated by the loess smooth curve (in orange), which highlights the increasing variance. The clear evidence of heteroscedasticity in microbiome count data underscores the need to account for this issue in the analysis.

To specifically address heteroscedasticity, or overdispersion, researchers have moved beyond the simpler Poisson distribution and considered more complex parametric models such as the negative binomial distribution or the generalized Poisson distribution (Qiao et al., 2024). These sophisticated models provide more robust inferences, capturing the variability and intrinsic heteroscedasticity in microbiome data more effectively. An alternative approach is the quasi-likelihood, which allows for flexible modeling of the variance by the mean of the data with a function, accommodating the inherent variability and improving the accuracy of differential abundance analysis (Wedderburn, 1974; McCullagh and Nelder, 1989; Nelder and Pregibon, 1987; Chen et al., 2013; Shi et al., 2023).

Over the past a few years, many differential abundance analysis methods have been developed to address specific characteristics for microbiome data (e.g., DESeq2, ALDEx2, and edgeR). We refer the readers to the recent review by Yang and Chen (2022), in which existing differential abundance analysis methods are evaluated through comprehensive simulation studies. Although modeling the count data directly seems a natural approach, those count-based parametric models face the challenge of inadequate false positive control, probably due to the model underfit for complex microbiome data. Thus, recent developments such as ANCOM-BC2 and LinDA are all based on transformed proportion data, where the variability associated with the count sampling process has not been accounted for (Yang and Chen, 2022). As most of the microbial taxa are of low abundance and their sampling variability is large, ignoring the sampling variability could reduce the statistical power to detect the differential abundance for these less abundant taxa. Therefore, the count-based models still hold the potential to be more powerful once the false positive control problem is fixed.

The main aim of our study is to propose simple yet effective methods to analyze microbiome abundance count data. We present an alternative framework to address heteroscedasticity in microbiome data. We utilize a Poisson distribution to obtain parameter estimates in regression models for microbiome counts. Although the Poisson distribution may be mis-specified, the resulting parameter estimates remain consistent, provided that the mean structure (the log linear model) is correctly specified. However, the standard error estimate for this parameter estimated from the mis-specified model (e.g., in the presence of heteroscedasticity) would be biased. We will consider two robust approaches to estimate the standard errors of the parameter of interest. The first approach involves using the bootstrap method to estimate its standard error. The advantage of this method lies in the straightforward parameter estimation under the Poisson distribution assumption, while its standard error is derived via the bootstrap method using the same assumption. This process helps estimate variability and construct confidence intervals for parameter estimates without relying on strict parametric assumptions (Efron and Tibshirani, 1994). The second method is the sandwich robust estimator, which offers reliable standard errors and confidence intervals for parameter estimates in statistical models. This is particularly useful when certain assumptions are not fully met (Cameron et al., 2007). Although the sandwich robust estimator is used to model the covariance structure for correlated data (LIANG and ZEGER, 1986), e.g., generalized estimating equations, its application to tackle heteroscedastic data is less common (Zeileis, 2006).

Through comprehensive simulation studies, we find that both the bootstrap and sandwich methods perform well in modeling microbiome differential abundance. The bootstrap method offers a flexible, data-driven approach to estimate variability, while the sandwich estimator provides a straightforward adjustment for heteroscedasticity. Together, these methods provide a robust framework for analyzing complex microbiome data, accommodating inherent variability, and improving the precision of statistical inferences. Our findings suggest that employing these covariance estimation methods can significantly improve the performance of microbiome differential abundance analyses.

The rest of the paper is organized as follows. Section 2 outlines the methodology, detailing the integration of two covariance estimation methods with Poisson regression to address heteroscedasticity in microbiome count data. Section 3 presents the simulation study results, comparing the performance of our proposed methods against other commonly used models under various conditions and distributions. Section 4 applies the proposed methods to two real data collected from human gut and vagina, respectively, highlighting their practical utility and effectiveness. Finally, Section 5 discusses the findings, implications for microbiome research, and directions for future work.

## 2 Methodology

In this section we will first present the Poisson (log linear) regression model, assuming a correct mean structure. We will then demonstrate the robust variance estimation methods.

### 2.1 Poisson regression mean structure

The mean structure of our proposed models is derived from Poisson regression, which has a simple structure commonly used for count data. In the context of microbiome abundance count data, the model is fitted for each taxon individually. For the taxon of interest, we denote its read count in sample i by $y_{i}$ , and $\mu_{i}=E\left(y_{i}\right)$ represents the mean of $y_{i}$ , for i=1,2,…,n . μ and y are the vector forms of $\mu_{i}$ and $y_{i}$ , respectively. $X_{i}$ is a p -dimensional vector representing the covariates for the i -th sample, and β is the corresponding coefficient vector. The mean structure is defined with a log link function as:

$$\log\left(\mu_{i}\right)=X_{i}^{T}\beta$$

As the mean structure of the Poisson regression is correct, we use the iterative reweighted least square (IRLS) method to yield the unbiased coefficient estimation. The initial values of IRLS are chosen from an intercept-only model, i.e., the initial value of the intercept is set to the log of the mean of the response variable, and the initial values for the coefficients of the predictors are set to zero. For Poisson regression, the weights matrix $W_{i}^{\left(k\right)}$ is a diagonal matrix with the elements of the vector $w_{i}^{\left(k\right)}=\mu_{i}^{\left(k\right)}=\exp\;\left(X_{i}^{T}\beta^{\left(k\right)}\right)$ , and zeros elsewhere. Here ${\hat{W}}_{i}^{\left(k\right)},{\hat{\mu }}_{i}^{\left(k\right)}$ and ${\hat{\beta }}^{\left(k\right)}$ are the corresponding estimation at each iteration k , and $X={\left(X_{1}^{T},X_{2}^{T},\ldots ,X_{n}^{T}\right)}^{T}$ is a matrix of dimension n×p . The coefficient estimates are updated as follows:

$${\hat{\beta }}^{\left(k+1\right)}={\left(X^{T}{\hat{W}}^{\left(k\right)}X\right)}^{‐1}X^{T}{\hat{W}}^{\left(k\right)}\left(y‐{\hat{\mu }}^{\left(k\right)}\right)$$

This iterative process continues until convergence, i.e., until the change in the coefficients β between iterations (measured using the Euclidean norm) is smaller than a specified tolerance level.

However, since the variance structure may differ from that of the Poisson distribution, the Poisson-based model will yield biased covariance estimate of the regression coefficients. Next, we propose two simplified methods for covariance estimation under such a mis-specified model.

### 2.2 Bootstrap method for covariance estimation

To obtain robust estimates of the covariance matrix $\hat{\Sigma }$ of the estimated Poisson regression coefficients $\hat{\beta }$ , we employ the bootstrap method, which involves resampling the data with replacement and refitting the model multiple times. By generating numerous simulated samples, the bootstrap method provides an empirical distribution of the estimated coefficients $\hat{\beta }$ , from which the covariance matrix $\hat{\Sigma }$ can be estimated (Efron and Tibshirani, 1994). This approach does not rely on the correct specification of the parametric distribution or the variance structure. The steps for using the bootstrap method are as follows:

Step 1: Resample the original dataset with replacement to create a bootstrap sample.
Step 2: Fit the Poisson regression model to the bootstrap sample.
Step 3: Repeat steps 1 and 2 multiple times (e.g., 1,000 iterations) to generate a distribution of the coefficient estimates.
Step 4: Calculate the covariance matrix $\hat{\Sigma }$ of the coefficient estimates $\hat{\beta }$ from the bootstrap samples.

These steps can be easily accomplished using the ‘boot’ package in R (Canty and Ripley, 2024; Davison and Hinkley, 1997).

### 2.3 Sandwich robust estimator

The sandwich robust estimator, also known as the heteroscedasticity-consistent (HC) estimator, provides reliable standard errors and confidence intervals for parameter estimates, even when model assumptions such as homoscedasticity are violated. The sandwich estimator is particularly useful in the context of small sample sizes or when the data exhibits heteroscedasticity. Considering the situation of small sample size in microbiome data, the Heteroscedasticity-consistent (HC) 3 estimators is recommended (Cameron et al., 2007). The HC3 estimator is designed for limited sample size, making it particularly suitable for microbiome studies, as it adjusts for potential heteroscedasticity by scaling the residuals in a way that is robust to small sample sizes. The sandwich Poisson regression is performed by the following steps:

Step 1: Fit the Poisson regression model to the original dataset.
Step 2: Calculate the residuals $\hat{e_{i}}$ , which is the i -th element of residual vector $\hat{e}=y-\exp\;X\hat{\beta }$ and leverage values $h_{ii}$ , which is the i -th diagonal element of hat matrix $H=X{\left(X^{T}WX\right)}^{-1}X^{T}W$ from the fitted model.
Step 3: Adjust the covariance matrix of the coefficient estimates using the HC3 formula, which incorporates the leverage values to correct for heteroscedasticity. The formula is given as:

$$\mathrm{Cov}\left(\hat{\beta }\right)={\left(X^{T}\text{WX}\right)}^{‐1}X^{T}\left[\mathrm{diag}{\left(\frac{\hat{e_{1}}}{1‐h_{11}},\ldots ,\frac{\hat{e_{i}}}{1‐h_{\mathrm{ii}}},\ldots ,\frac{\hat{e_{n}}}{1‐h_{\mathrm{nn}}}\right)}^{2}\right]X{\left(X^{T}\mathrm{WX}\right)}^{‐1}$$

where $diag\left(a\right)$ denotes a diagonal matrix with the elements of the vector a on its diagonal, and zeros elsewhere. Then W is the weight matrix of Poisson IRLS estimation, defined as the diagonal matrix $diag\left(w_{1},\ldots ,w_{i},\ldots ,w_{n}\right)$ , and $w_{i}=\mu_{i}=\exp\;\left(X_{i}^{T}\hat{\beta }\right)$ .

These steps are implemented by using the ‘sandwich’ package in R program (Zeileis et al., 2020).

## 3 Simulation study

In this section, we present comprehensive simulation studies to compare our proposed models with other widely used alternative methods. The existing models selected for comparison include negative binomial regression, Poisson regression, Generalized Poisson (GP) regression, Quasi-Poisson (QP) regression. The negative binomial regression and Poisson regression are generalized linear models which assumes the data following specific parametric distributions; they serve as the baseline models for comparison.

The generalized Poisson (GP) regression and Quasi-Poisson (QP) regression are commonly used to address overdispersion. The generalized Poisson regression, also known as two-parameter generalized Poisson distribution, was proposed by Consul and Jain (1973) (Consul and Jain, 1973). The GP distribution’s probability mass function is defined as:

$$f\left(y\right)=\frac{\theta {\left(\theta +\lambda y\right)}^{y‐1}\exp\left(‐\theta ‐\lambda y\right)}{y!}$$

where θ > 0 , 0<λ < 1 and y=0,1,2... . The mean of GP distribution is $\mu =E\left(Y\right)=\frac{\theta }{1-\lambda }$ and the variance of GP distribution is $\left(Y\right)=\frac{\theta }{{\left(1-\lambda \right)}^{3}}$ , so it has a mean variance function as $Var\left(Y\right)=\frac{\mu }{{\left(1-\lambda \right)}^{2}}$ . Three variants of the GP model are available, using different parameterizations. The original parameterization, referred to as the GP-0 in the R package ‘VGAM’, serves as the baseline of GP models. The GP-1 and GP-2 are proposed by Yang et al. (2009) offering alternative parameterizations more suitable for regression. The GP-1 set $\theta =\frac{\mu }{\sqrt{\varphi }}$ and $\lambda =1-\frac{1}{\sqrt{\varphi }}$ , where μ is the mean and φ>0 , resulting in a variance of φμ . The GP-2 has $\theta =\frac{\mu }{1+\alpha \mu }$ and $\lambda =\frac{\alpha \mu }{1+\alpha \mu }$ where μ is the mean and $>\min\left(-\frac{1}{\mu },-\frac{1}{y}\right)$ , yielding a variance as ${\left(1+\alpha \mu \right)}^{2}\mu$ . (Zeileis et al., 2020) However, the GP-0 and GP-1 models demonstrate suboptimal performance under conditions of under-dispersion, as observed in our simulation studies involving Gamma and Pareto distributions (results available upon request). This issue aligns with findings in the literature, such as those discussed by Scollnik (1998). Specifically, when the parameter λ of the GP distribution is negative, the probability mass function is no longer normalized—meaning that the sum of the probabilities does not equal 1—resulting in poor model performance. In contrast, the GP-2 model, which allows for α<0 , effectively manages under-dispersion. Consequently, we focus exclusively on GP-2 regression in subsequent analyses.

The Quasi-Poisson (QP) regression model is an extension of the Poisson regression model widely used to address overdispersion. The model assumes that the variance is a linear function of the mean, $V\left(Y\right)=\phi \mu$ , where ϕ is the dispersion parameter. The estimation is performed using maximum quasi-likelihood estimation.

### 3.1 Data generated from various distributions (under model misspecifications)

We design a simulation study to evaluate the performance of our models under model misspecifications with data generated from various distributions. Each simulation dataset has a sample size of 200 and includes a count variable Y along with a univariate covariate X . The variable X is a binary variable with 50% of observations being 0 and 50% being 1. The count variable Y is generated following different distributions but shares the same mean structure as $E\left(Y\right)=\mu =\exp\;\left(\beta_{0}+\beta_{1}X\right)$ . The simulation process is repeated 500 times. We compare these models on data generated from various distributions, including the Gamma distribution, Poisson distribution, Pareto distribution, Over-dispersed Poisson distribution and Negative Binomial distribution. These distributions have different mean-variance relationships, allowing us to comprehensively evaluate the proposed models’ ability to handle model misspecification.

#### 3.1.1 Distributions

##### 3.1.1.1 Mis-specified Gamma distribution

The simulation datasets are generated following $Y\;∼\text{ Gamma }\left(k,\theta \right)$ , where $k=\mu^{1.5}$ and $\theta =\mu^{-0.5}$ . The density function of the Gamma distribution is given by $f\left(x;k,\theta \right)=\frac{x^{k-1}e^{\frac{-x}{\theta }}}{\theta^{k}\;\Gamma \left(k\right)}$ , where x>0 is the variable, k>0 is the shape parameter and θ>0 is the scale parameter. $\Gamma \left(k\right)$ is the Gamma function defined as $\Gamma \left(k\right)=\int_{0}^{\infty }t^{k-1}e^{-t}dt$ . Therefore, the mean and variance are $E\left(Y\right)=k\theta =\mu$ and $Var\left(Y\right)=k\theta^{2}=\mu^{0.5}$ , respectively. The data are generated using the R function ‘rpois’ from the ‘stats’ package.

##### 3.1.1.2 Poisson distribution

The simulation datasets are generated following $Y\;∼\text{ Poisson }\left(\lambda \right)$ , where the mean and variance are $E\left(Y\right)=Var\left(Y\right)=\lambda$ . The data are generated using the R function ‘rpois’ from the ‘stats’ package.

##### 3.1.1.3 Pareto distribution

The Pareto distribution is a power-law distribution originally designed to describe the distribution of wealth in a society, fitting the phenomena of “80–20” rule that 80% of wealth is held by a small fraction of the population (Gabaix, 2009). This distribution is analogous to the characteristics observed in microbiome count data.

Here we consider a Pareto (Type I) distribution, and the simulation datasets are generated following $Y\;∼\text{ Pareto }\left(\gamma ,x_{m}\right)$ , where γ=5 , and $x_{m}=\frac{4}{5}\mu$ . The density function of Pareto Type I distribution is given by $f\left(x;\gamma ,x_{m}\right)=\frac{\gamma {x_{m}}^{\gamma }}{x^{\gamma +1}}$ , where α>0 is the shape parameter, $x_{m}>0$ is the scale parameter, and $x\geq x_{m}$ . In this form, $x_{m}$ is the minimum value that x can take, and α controls the “heaviness” of the tail of the distribution. Therefore, the mean and variance are $E\left(Y\right)=\frac{\gamma x_{m}}{\gamma -1}=\frac{5}{4}x_{m}=\mu$ and $Var\left(Y\right)=\frac{\gamma {x_{m}}^{2}}{{\left(\gamma -1\right)}^{2}\left(\gamma -2\right)}={\frac{5}{48}x_{m}}^{2}=\frac{1}{15}\mu^{2}$ . The data are generated using the R function ‘rpareto’ from the ‘VGAM’ package.

##### 3.1.1.4 Negative binomial distribution

The Negative Binomial distribution is used for the number of trials needed to achieve a specified number of successes in a sequence of independent and identically distributed Bernoulli trials. The density function of a Negative Binomial distribution with number of failures r and success probability p ( 0<p≤1 ) is $\frac{\Gamma \left(y+r\right)}{\Gamma \left(r\right)y!}p^{r}{\left(1-p\right)}^{y}$ for y=0,1,2,… The parameterization is determined as the mean $E\left(Y\right)=\mu =\frac{r\left(1-p\right)}{p}$ and the variance $Var\left(Y\right)=\frac{r\left(1-p\right)}{p^{2}}$ . Therefore, the mean variance function as $Var\left(Y\right)=\frac{\mu^{2}}{r}+\mu$ . Here we set r=0.1 , resulting in $Var\left(Y\right)=10\mu^{2}+\mu$ , to mimic the high over-dispersion observed in certain taxa (e.g., *Lactobacillus*) in the real data in Section 4. The data are generated using the R function ‘rnbinom’ from the ‘stats’ package.

##### 3.1.1.5 Over-dispersed Poisson distribution

The simulation datasets are generated by a mis-specified negative binomial distribution with $E\left(Y\right)=\mu$ and $Var\left(Y\right)=2\mu$ . The data are also generated using the R function ‘rnbinom’ from the ‘stats’ package with the variance set to 2μ .

We aimed to evaluate the models’ performance based on their ability to control type I error and their power in detecting true positives. We will evaluate their ability to estimate the variance of coefficients by assessing the closeness of the sampling mean of the standard error estimate (SEM) to the standard error of the estimate (SEE). Additionally, we consider metrics including bias, mean squared error (MSE), and coverage probability (CP):

• Bias measures the difference between the average of the parameter estimates and the true parameter value: $Bias=\frac{\sum_{i=1}^{N}\left({\hat{\omega }}_{i}-\omega \right)}{N}$ , where ${\hat{\omega }}_{i}$ is the parameter estimate for the i th replicate, ω is the true value of the parameter in the simulation study, and N is the total number of replicates;
• SEE represents the empirical standard error of the parameter estimate, calculated as: $SEE=\sqrt{\frac{\sum_{i=1}^{N}{\left({\hat{\omega }}_{i}-\bar{\omega }\right)}^{2}}{N-1}}$ , where ${\hat{\omega }}_{i}$ is the parameter estimate for the i th replicate and $\bar{\omega }$ is the average of the parameter estimates ${\hat{\omega }}_{i}$ ’s in the simulation study;
• SEM is the mean of the standard error estimates across all replicates: $SEM=\frac{\sum_{i=1}^{N}{SE}_{i}}{N}$ , where ${SE}_{i}$ is the standard error estimate for the i th replicate;
• MSE is the average of the squared differences between the estimated parameters and the true parameter value, calculated as: $\frac{\sum_{i=1}^{N}{\left({\hat{\omega }}_{i}-\omega \right)}^{2}}{N}$ ;
• CP is defined as the proportion of simulation runs in which the true parameter value lies within the estimated confidence interval. The formula is: $CP=\frac{1}{N}\sum_{i=1}^{N}I\left(\omega \in \left[{\hat{\omega }}_{i}^{lower},{\hat{\omega }}_{i}^{upper}\right]\right)$ , where $I\left(∙\right)$ is the indicator function that equals 1 if the true parameter ω is within the confidence interval $\left[{\hat{\omega }}_{i}^{lower},{\hat{\omega }}_{i}^{upper}\right]$ for the i th simulation, and 0 otherwise. The desired coverage probability is close to the nominal level - 95% in our study, meaning that we expect the confidence intervals to contain the true parameter value 95% of the time. If the CP is much higher than 95%, it suggests that the confidence intervals are too wide, leading to overly conservative inferences. If the CP is substantially lower than 95%, it indicates that the confidence intervals are too narrow, which increases the risk of not capturing the true parameter value, leading to misleading inferences.

In simulation setting 1 we set $\beta_{0}=2$ and $\beta_{1}=0$ , ensuring no association between X and Y . This allows us to evaluate the Type I error rate for all proposed models. In simulation setting 2 we set $\beta_{0}=2$ and $\beta_{1}=0.1$ , corresponding to a small effect. In simulation setting 3 we set $\beta_{0}=2$ and $\beta_{1}=1.5$ , corresponding to a large effect. The settings allow us to assess how well the models perform in detecting different effect sizes ( $\beta_{1}=0.1$ and $\beta_{1}=1.5$ ) between X and Y .

The results of simulation settings 1-3 are presented in Tables 1–15, respectively. We first observe that all methods have small biases in parameter estimates of $\beta_{0}$ and $\beta_{1}$ , as all of them have correct specification of the mean. Our focuses are on (i) whether the standard error estimates (SEM) for ${\hat{\beta }}_{0}$ and ${\hat{\beta }}_{1}$ are close to the empirical standard deviation (SEE); (ii) whether the coverage probabilities for ${\hat{\beta }}_{0}$ and ${\hat{\beta }}_{1}$ are close to the nominal level 0.95; (iii) whether the Type I errors in Tables 1–5 are well controlled (close to 0.05); (iv) the powers in Tables 6–10 under well controlled Type I errors in Tables 1–5. Of note, due to the large value of $\beta_{1}$ , most of the powers in Tables 11–15 are 1.

**TABLE 1:** Simulation setting 1 with no effect. True model: Gamma distribution.

Parameter	Metric	Negative Binomial	Poisson	Bootstrap	Sandwich	Generalized Poisson - 2	Quasi-Poisson
$\beta_{0}$	Bias	0.000179	0.000179	0.000179	0.000179	0.000179	0.000179
	SEE	0.0205	0.0205	0.0205	0.0205	0.0205	0.0205
	SEM	0.0368	0.0368	0.0220	0.0222	0.0220	0.0222
	MSE	0.000421	0.000421	0.000421	0.000421	0.000421	0.000421
	CP	1.000	1.000	0.964	0.966	0.964	0.966
$\beta_{1}$	Bias	0.00105	0.00105	0.00105	0.00105	0.00105	0.00105
	SEE	0.0291	0.0291	0.0291	0.0291	0.0291	0.0291
	SEM	0.0520	0.0520	0.0313	0.0315	0.0312	0.0315
	MSE	0.000846	0.000846	0.000846	0.000846	0.000846	0.000846
	CP	0.996	0.996	0.952	0.952	0.954	0.952
	Type I error	0.004	0.004	0.048	0.048	0.046	0.048

**TABLE 2:** Simulation setting 1 with no effect. True model: Poisson distribution.

Parameter	Metric	Negative Binomial	Poisson	Bootstrap	Sandwich	Generalized Poisson - 2	Quasi-Poisson
$\beta_{0}$	Bias	0.00133	0.00133	0.00138	0.00138	0.00133	0.00133
	SEE	0.0362	0.0362	0.0362	0.0362	0.0362	0.0362
	SEM	0.0375	0.0368	0.0366	0.0369	0.0367	0.0369
	MSE	0.00131	0.00131	0.00131	0.00131	0.00131	0.00131
	CP	0.954	0.954	0.950	0.954	0.954	0.954
$\beta_{1}$	Bias	0.00022	0.00022	0.00022	0.00022	0.00022	0.00022
	SEE	0.0522	0.0522	0.0522	0.0522	0.0522	0.0522
	SEM	0.0530	0.0521	0.0517	0.0523	0.0519	0.0523
	MSE	0.00272	0.00272	0.00272	0.00272	0.00272	0.00272
	CP	0.962	0.956	0.950	0.958	0.952	0.958
	Type I error	0.038	0.044	0.050	0.042	0.048	0.042

**TABLE 3:** Simulation setting 1 with no effect. True model: Pareto distribution.

Parameter	Metric	Negative Binomial	Poisson	Bootstrap	Sandwich	Generalized Poisson - 2	Quasi-Poisson
$\beta_{0}$	Bias	0.00207	0.00207	0.00207	0.00207	0.00207	0.00207
	SEE	0.0261	0.0261	0.0261	0.0261	0.0261	0.0261
	SEM	0.0369	0.0368	0.0241	0.0244	0.0241	0.0244
	MSE	0.00069	0.00069	0.00069	0.00069	0.00069	0.00069
	CP	0.988	0.986	0.912	0.910	0.906	0.910
$\beta_{1}$	Bias	0.00159	0.00159	0.00159	0.00159	0.00159	0.00159
	SEE	0.0352	0.0352	0.0352	0.0352	0.0352	0.0352
	SEM	0.0521	0.0521	0.0347	0.0351	0.0347	0.0351
	MSE	0.00124	0.00124	0.00124	0.00124	0.00124	0.00124
	CP	0.992	0.990	0.946	0.946	0.944	0.946
	Type I error	0.008	0.010	0.054	0.054	0.056	0.054

**TABLE 4:** Simulation setting 1 with no effect. True model: Over-dispersed Poisson.

Parameter	Metric	Negative Binomial	Poisson	Bootstrap	Sandwich	Generalized Poisson - 2	Quasi-Poisson
$\beta_{0}$	Bias	0.000218	0.000218	0.000218	0.000218	0.000218	0.000218
	SEE	0.0508	0.0508	0.0508	0.0508	0.0508	0.0508
	SEM	0.0517	0.0368	0.0515	0.0519	0.0516	0.0519
	MSE	0.00257	0.00257	0.00257	0.00257	0.00257	0.00257
	CP	0.954	0.842	0.948	0.950	0.948	0.95
$\beta_{1}$	Bias	9.14E-05	9.14E-05	9.14E-05	9.14E-05	9.14E-05	9.14E-05
	SEE	0.0746	0.0746	0.0746	0.0746	0.0746	0.0746
	SEM	0.0731	0.0521	0.0733	0.0738	0.0734	0.0738
	MSE	0.00555	0.00555	0.00555	0.00555	0.00555	0.00555
	CP	0.946	0.838	0.944	0.946	0.946	0.946
	Type I error	0.054	0.162	0.056	0.054	0.054	0.054

**TABLE 5:** Simulation setting 1 with no effect. True model: Negative binomial.

Parameter	Metric	Negative Binomial	Poisson	Bootstrap	Sandwich	Generalized Poisson - 2	Quasi-Poisson
$\beta_{0}$	Bias	0.0374	0.0374	0.0374	0.0374	0.0374	0.0374
	SEE	0.325	0.325	0.325	0.325	0.325	0.325
	SEM	0.317	0.0380	0.311	0.298	0.584	0.305
	MSE	0.107	0.107	0.107	0.107	0.107	0.107
	CP	0.950	0.170	0.934	0.924	0.992	0.938
$\beta_{1}$	Bias	0.0322	0.0322	0.0322	0.0322	0.0322	0.0322
	SEE	0.455	0.455	0.455	0.455	0.455	0.455
	SEM	0.448	0.0545	0.448	0.426	0.824	0.437
	MSE	0.208	0.208	0.208	0.208	0.208	0.208
	CP	0.958	0.170	0.954	0.944	1.000	0.966
	Type I error	0.042	0.830	0.046	0.056	0.000	0.034

**TABLE 6:** Simulation setting 2 with small effect ( $\beta_{1}=0.1$ ). True model: Gamma distribution.

Parameter	Metric	Negative Binomial	Poisson	Bootstrap	Sandwich	Generalized Poisson - 2	Quasi-Poisson
$\beta_{0}$	Bias	0.00018	0.00018	0.00018	0.00018	0.00018	0.00018
	SEE	0.0205	0.0205	0.0205	0.0205	0.0205	0.0205
	SEM	0.0368	0.0368	0.0220	0.0222	0.0220	0.0222
	MSE	0.0004	0.0004	0.0004	0.0004	0.0004	0.0004
	CP	1.00	1.00	0.968	0.966	0.964	0.966
$\beta_{1}$	Bias	0.00060	0.00060	0.00060	0.00060	0.00060	0.00060
	SEE	0.0280	0.0280	0.0280	0.0280	0.0280	0.0280
	SEM	0.0508	0.0508	0.0303	0.0304	0.030	0.030
	MSE	0.00078	0.00078	0.00078	0.00078	0.00078	0.00078
	CP	0.996	0.996	0.950	0.952	0.952	0.952
	power	0.504	0.504	0.928	0.924	0.926	0.924

**TABLE 7:** Simulation setting 2 with small effect ( $\beta_{1}=0.1$ ). True model: Poisson distribution.

Parameter	Metric	Negative Binomial	Poisson	Bootstrap	Sandwich	Generalized Poisson - 2	Quasi-Poisson
$\beta_{0}$	Bias	0.00027	0.00027	0.00027	0.00027	0.00027	0.00027
	SEE	0.0388	0.0388	0.0388	0.0388	0.0388	0.0388
	SEM	0.0374	0.0368	0.0364	0.0367	0.0364	0.0367
	MSE	0.00150	0.00150	0.00150	0.00150	0.00150	0.00150
	CP	0.942	0.940	0.934	0.932	0.928	0.932
$\beta_{1}$	Bias	0.00104	0.00104	0.00104	0.00104	0.00104	0.00104
	SEE	0.0520	0.0520	0.0520	0.0520	0.0520	0.0520
	SEM	0.0516	0.0508	0.0505	0.0508	0.0505	0.0508
	MSE	0.00270	0.00270	0.00270	0.00270	0.00270	0.00270
	CP	0.946	0.942	0.938	0.938	0.934	0.938
	power	0.494	0.508	0.504	0.502	0.516	0.502

**TABLE 8:** Simulation setting 2 with small effect ( $\beta_{1}=0.1$ ). True model: Pareto distribution.

Parameter	Metric	Negative Binomial	Poisson	Bootstrap	Sandwich	Generalized Poisson - 2	Quasi-Poisson
$\beta_{0}$	Bias	0.00109	0.00109	0.00109	0.00109	0.00235	0.00109
	SEE	0.0255	0.0255	0.0255	0.0255	0.0262	0.0255
	SEM	0.0368	0.0368	0.0249	0.0252	0.0369	0.0259
	MSE	0.00065	0.00065	0.00065	0.00065	0.00069	0.00065
	CP	0.990	0.990	0.938	0.942	0.990	0.950
$\beta_{1}$	Bias	0.00080	0.00080	0.00080	0.00080	0.00894	0.00080
	SEE	0.0371	0.0371	0.0371	0.0371	0.0374	0.0371
	SEM	0.0509	0.0508	0.0353	0.0358	0.0508	0.0358
	MSE	0.00137	0.00137	0.00137	0.00137	0.00148	0.00137
	CP	0.984	0.980	0.946	0.950	0.982	0.948
	power	0.488	0.488	0.810	0.804	0.606	0.808

**TABLE 9:** Simulation setting 2 with small effect ( $\beta_{1}=0.1$ ). True model: Over-dispersed Poisson distribution.

Parameter	Metric	Negative Binomial	Poisson	Bootstrap	Sandwich	Generalized Poisson - 2	Quasi-Poisson
$\beta_{0}$	Bias	0.00022	0.00022	0.00022	0.00020	0.00022	0.00022
	SEE	0.0508	0.0508	0.0508	0.0508	0.0508	0.0508
	SEM	0.0511	0.0368	0.0516	0.0519	0.0517	0.0519
	MSE	0.00257	0.00257	0.00257	0.00258	0.00257	0.00257
	CP	0.946	0.842	0.944	0.950	0.946	0.950
$\beta_{1}$	Bias	0.00046	0.00046	0.00046	0.00027	0.00046	0.00046
	SEE	0.0703	0.0703	0.0703	0.0702	0.0703	0.0703
	SEM	0.0713	0.0508	0.0715	0.0720	0.0716	0.0720
	MSE	0.00493	0.00493	0.00493	0.00492	0.00493	0.00493
	CP	0.950	0.830	0.954	0.958	0.956	0.958
	power	0.294	0.504	0.290	0.287	0.296	0.286

**TABLE 10:** Simulation setting 2 with small effect ( $\beta_{1}=0.1$ ). True model: Negative binomial distribution.

Parameter	Metric	Negative Binomial	Poisson	Bootstrap	Sandwich	Generalized Poisson - 2	Quasi-Poisson
$\beta_{0}$	Bias	0.0374	0.0374	0.0374	0.0374	0.0374	0.0374
	SEE	0.325	0.325	0.325	0.325	0.325	0.325
	SEM	0.317	0.038	0.311	0.298	0.580	0.312
	MSE	0.107	0.107	0.107	0.107	0.107	0.107
	CP	0.948	0.170	0.934	0.924	0.990	0.938
$\beta_{1}$	Bias	0.0364	0.0364	0.0364	0.0364	0.0364	0.0364
	SEE	0.459	0.459	0.459	0.459	0.459	0.459
	SEM	0.448	0.0532	0.444	0.427	0.835	0.438
	MSE	0.211	0.211	0.211	0.211	0.211	0.211
	CP	0.950	0.170	0.948	0.932	1.00	0.962
	power	0.064	0.826	0.058	0.068	0	0.044

**TABLE 11:** Simulation setting 3 with large effect ( $\beta_{1}=1.5$ ). True model: Gamma distribution.

Parameter	Metric	Negative Binomial	Poisson	Bootstrap	Sandwich	Generalized Poisson - 2	Quasi-Poisson
$\beta_{0}$	Bias	0.00018	0.00018	0.00018	0.00018	0.00035	0.00018
	SEE	0.0205	0.0205	0.0205	0.0205	0.0209	0.0205
	SEM	0.0368	0.0368	0.0220	0.0221	0.0368	0.0190
	MSE	0.00042	0.00042	0.00042	0.00042	0.00044	0.00042
	CP	1.000	1.000	0.964	0.966	1.000	0.932
$\beta_{1}$	Bias	0.00004	0.00004	0.00004	0.00004	0.00023	0.00004
	SEE	0.0212	0.0212	0.0212	0.0212	0.0216	0.0212
	SEM	0.0407	0.0407	0.0232	0.0232	0.0407	0.0210
	MSE	0.00045	0.00045	0.00045	0.00045	0.00047	0.00045
	CP	0.998	0.998	0.962	0.964	0.998	0.934
	power	1.000	1.000	1.000	1.000	1.000	1.000

**TABLE 12:** Simulation setting 3 with large effect ( $\beta_{1}=1.5$ ). True model: Poisson distribution.

Parameter	Metric	Negative Binomial	Poisson	Bootstrap	Sandwich	Generalized Poisson - 2	Quasi-Poisson
$\beta_{0}$	Bias	0.00133	0.00133	0.00133	0.00133	0.00133	0.00133
	SEE	0.0362	0.0362	0.0362	0.0362	0.0362	0.0362
	SEM	0.0370	0.0368	0.0366	0.0368	0.0370	0.0368
	MSE	0.00131	0.00131	0.00131	0.00131	0.00131	0.00131
	CP	0.954	0.954	0.950	0.954	0.954	0.954
$\beta_{1}$	Bias	0.00259	0.00259	0.00259	0.00259	0.00259	0.00259
	SEE	0.0402	0.0402	0.0402	0.0402	0.0402	0.0402
	SEM	0.0411	0.0407	0.0406	0.0407	0.0411	0.0407
	MSE	0.00162	0.00162	0.00162	0.00162	0.00162	0.00162
	CP	0.958	0.954	0.952	0.950	0.958	0.954
	power	1.000	1.000	1.000	1.000	1.000	1.000

**TABLE 13:** Simulation setting 3 with large effect ( $\beta_{1}=1.5$ ). True model: Pareto distribution.

Parameter	Metric	Negative Binomial	Poisson	Bootstrap	Sandwich	Generalized Poisson - 2	Quasi-Poisson
$\beta_{0}$	Bias	0.00207	0.00207	0.00207	0.00207	0.00615	0.00207
	SEE	0.0261	0.0261	0.0261	0.0261	0.0272	0.0261
	SEM	0.0388	0.0368	0.0241	0.0243	0.0388	0.0410
	MSE	0.00069	0.00069	0.00069	0.00069	0.00078	0.00069
	CP	0.992	0.986	0.912	0.910	0.992	0.996
$\beta_{1}$	Bias	0.00159	0.00159	0.00159	0.00159	0.00610	0.00159
	SEE	0.0352	0.0352	0.0352	0.0352	0.0358	0.0352
	SEM	0.0442	0.0407	0.0347	0.0349	0.0444	0.0453
	MSE	0.00124	0.00124	0.00124	0.00124	0.00132	0.00124
	CP	0.986	0.976	0.944	0.946	0.986	0.978
	power	1.000	1.000	1.000	1.000	1.000	1.000

**TABLE 14:** Simulation setting 3 with large effect ( $\beta_{1}=1.5$ ). True model: Over-dispersed Poisson distribution.

Parameter	Metric	Negative Binomial	Poisson	Bootstrap	Sandwich	Generalized Poisson - 2	Quasi-Poisson
$\beta_{0}$	Bias	0.00022	0.00022	0.00022	0.00022	0.00022	0.00022
	SEE	0.0508	0.0508	0.0508	0.0508	0.0508	0.0508
	SEM	0.0424	0.0368	0.0515	0.0517	0.0413	0.0519
	MSE	0.00257	0.00257	0.00257	0.00257	0.00257	0.00257
	CP	0.906	0.842	0.944	0.950	0.890	0.954
$\beta_{1}$	Bias	0.00028	0.00028	0.00028	0.00028	0.00028	0.00028
	SEE	0.0562	0.0562	0.0562	0.0562	0.0562	0.0562
	SEM	0.0504	0.0407	0.0571	0.0573	0.0493	0.0574
	MSE	0.00315	0.00315	0.00315	0.00315	0.00315	0.00315
	CP	0.922	0.838	0.948	0.950	0.914	0.952
	power	1.000	1.000	1.000	1.000	1.000	1.000

**TABLE 15:** Simulation setting 3 with large effect ( $\beta_{1}=1.5$ ). True model: Negative binomial distribution.

Parameter	Metric	Negative Binomial	Poisson	Bootstrap	Sandwich	Generalized Poisson - 2	Quasi-Poisson
$\beta_{0}$	Bias	0.0389	0.0389	0.0389	0.0389	0.0389	0.0389
	SEE	0.334	0.334	0.334	0.334	0.334	0.334
	SEM	0.316	0.0380	0.312	0.299	0.499	0.501
	MSE	0.113	0.113	0.113	0.113	0.113	0.113
	CP	0.930	0.186	0.934	0.912	0.974	0.994
$\beta_{1}$	Bias	0.0243	0.0243	0.0243	0.0243	0.0243	0.0243
	SEE	0.443	0.443	0.443	0.443	0.443	0.443
	SEM	0.446	0.0423	0.442	0.425	1.10	0.556
	MSE	0.196	0.196	0.196	0.196	0.196	0.196
	CP	0.946	0.150	0.938	0.932	1.00	0.960
	power	0.910	1.00	0.918	0.922	0.0580	0.868

We note that the proposed models—the Bootstrap Poisson model and the Sandwich Poisson model have satisfactory performance across various forms of model misspecification. In Tables 1–15, the standard error estimates (SEM) for ${\hat{\beta }}_{0}$ and ${\hat{\beta }}_{1}$ are close to the empirical standard deviation (SEE), and the coverage probabilities for ${\hat{\beta }}_{0}$ and ${\hat{\beta }}_{1}$ are close to the nominal level 0.95. The Type I errors in Tables 1–5 are well controlled (close to 0.05), and the powers in Tables 6–10 are among the highest under well controlled Type I errors.

In contrast, the Poisson and negative binomial regression models tend to either under- or over-estimate the standard deviation when these assumptions are violated, which adversely affects their Type I error control and 95% coverage probability. For example, the standard deviations are over-estimated for Gamma and Pareto distributions (Tables 1, 3), resulting in conservative Type I error controls (small values in Tables 1, 3) and lower power in Tables 6, 8. On the other hand, the Poisson regression model under-estimates the standard deviation for over-dispersed Poisson and negative binomial distributions (Tables 4, 5), resulting in inflated Type I errors (potential false positives).

The Quasi-Poisson (QP) model performs relatively well in simulation settings 1 and 2. In particular, when dealing with small effect sizes $\beta_{1}=0.1$ (as in simulation setting 2 where $\mu =\exp\left(\beta_{0}+\beta_{1}X\right)$ has a relatively small range), the estimated coefficient standard errors, which are a linear function of the mean estimates ( $V\left(Y\right)=\phi \mu$ ), are close to the true values. However, in simulation setting 3, when the effect size $\beta_{1}$ increases to 1.5 (resulting in a larger range of μ ), the linear relationship $V\left(Y\right)=\phi \mu$ in the QP model poorly approximates the Pareto distribution ( $V\left(Y\right)=\frac{\mu^{2}}{15}$ ) and the Negative Binomial distribution ( $V\left(Y\right)=10\mu^{2}+\mu$ ). Consequently, this leads to much higher standard error estimates (SEM) for ${\hat{\beta }}_{0}$ and ${\hat{\beta }}_{1}$ than the empirical standard deviation (SEE), shown in Tables 13, 15. The coverage probabilities are thus much larger than 95%. Besides, these overestimated SEM values also lead to wider confidence intervals, which in turn reduces the statistical power for the QP model as shown in Table 15.

The Generalized Poisson-2 regression model, with variance defined as ${\left(1+\alpha \mu \right)}^{2}\mu$ , performs worse than the Quasi-Poisson model. Specifically, the GP-2 model exhibits poor performance when fitting data generated from a negative binomial distribution (Tables 5, 10, 15). For instance, Table 5 shows overly conservative Type I error control, while Table 10 reports low power. Additionally, the GP-2 model produces less satisfactory results for Pareto-distributed data (Tables 3, 8), Gamma-distributed data (Table 11), and over-dispersed Poisson data (Table 14).

### 3.2 Data generated from the ZicoSeq algorithm

In order to assess the ability of the models to handle the complex nature of microbiome data, we perform another simulation using the ZicoSeq algorithm (Yang and Chen, 2022). ZicoSeq is a combination of permutation-based simulation dataset generation algorithm and differential analysis method. It takes real microbiome datasets to generate simulation data which mimic the unique characteristics of microbiome datasets, such as high dimensionality, sparsity, zero inflation, and overdispersion. Since Yang and Chen’s review (Yang and Chen, 2022) has already established ZicoSeq’s superiority over other existing methods, we focus on comparing our model with ZicoSeq rather than these methods. Given the similarity between the GP-0, GP-1, and GP-2 models, and considering the superior performance of GP-2 in handling under-dispersion, we exclude the GP-0 and GP-1 models in the following studies and include only the GP-2 model as a representative of the Generalized Poisson methods. Additionally, we include two more recently developed and widely used microbiome differential analysis methods: LinDA (Zhou et al., 2022) and ANCOM-BC2 (Lin and Peddada, 2024) (a 2024 update of ANCOM-BC). The simulation study will help us assess our model’s effectiveness in the context of current leading methods. LinDA (Linear Models for Differential Abundance Analysis) is a differential abundance analysis method for microbiome data, using traditional linear models with center log ratio (CLR) transformed abundance data and a bias correction procedure to account for the compositional structure of microbiome data and employs regularization techniques to improve the robustness and accuracy of differential abundance detection. ANCOM-BC2 (Analysis of Composition of Microbiomes with Bias Correction-2) is an extension of the ANCOM-BC method, designed to identify differentially abundant taxa while controlling for the compositional bias. ANCOM-BC2 incorporates bias correction techniques to improve the accuracy and reliability of the differential abundance analysis in microbiome studies. The following simulation study is conducted using the most recent versions of each method’s respective package at the time of analysis: ZicoSeq (“Gunifrac” 1.8), LinDA (“MicrobiomeStat” 1.2), and ANCOM-BC2 (“ANCOMBC” 2.6.0).

We use the adenomas dataset as the reference dataset for ZicoSeq simulation dataset generation. Adenomas study focuses on the gut microbiota composition in patients with and without adenomatous polyps, precursors to colorectal cancer. The study generated 16S rRNA gene sequences from fecal samples of 266 patients with adenomas and 534 controls collected from standard screening colonoscopy operating between 2001 and 2005 at multiple medical centers (Hale et al., 2017). Following Shi et al. (2023), for the preprocessing we exclude operational taxonomic units (OTUs) with prevalence less than 25%, classifying OTUs with prevalence from 100% to 62.5% as common, and those with prevalence from 62.5% to 25% as rare. In our simulation, the covariate X is generated uniformly from 0 to 1. To explore the performance of the methods under different signal densities, we include two simulation settings: low (10%) and high (30%) signal density scenarios. In the low signal density scenario, 10% of the taxa Y are associated with X , while the remaining 90% have no association with X . In the high signal density scenario, 30% of the taxa Y are associated with X . For the differentially abundant taxa group, the abundance for taxon i is defined as: $Y_{i}=C_{i}\exp\left(0.2X_{i}+\epsilon_{i}\right)$ , where the random abundance $C_{i}$ is drawn from the reference adenomas dataset. We generate 400 samples and 100 taxa for each iteration, repeating this process 100 times. Performance is evaluated based on the average false discovery rate (FDR), true positive rate (TPR), F1 score, and Matthews Correlation Coefficient (MCC). The formulas for these metrics are as follows:

$$\text{False Discovery Rate }\left(\text{FDR}\right)=\frac{\text{False}\;\text{Positives}\;\left(\text{FP}\right)}{\text{False}\;\text{Positives}\left(\text{FP}\right)+\text{True}\;\text{Positives}\left(\text{TP}\right)}$$

$$\text{True Positive Rate }\left(\text{TPR}\right)=\frac{\text{True}\;\text{Positives}\;\left(\text{TP}\right)}{\text{False}\;\text{Negatives}\left(\text{FN}\right)+\text{True}\;\text{Positives}\left(\text{TP}\right)}$$

$$F1\text{ score}=\frac{2\times \text{Precision}\times \text{Recall}}{\text{Precision}+\text{Recall}},\text{where }\text{Precision}=\frac{\text{TP}}{\text{TP}+\text{FP}}\text{ and Recall}=\text{TPR}$$

$$\text{Matthews Correlation Coefficient }\left(\text{MCC}\right)=\frac{\text{TP}\times \text{TN}‐\text{FP}\times \text{FN}}{\sqrt{\left(\text{TP}+\text{FP}\right)\left(\text{TP}+\text{FN}\right)\left(\text{TN}+\text{FP}\right)\left(\text{TN}+\text{FN}\right)}}$$

**FIGURE 2:**
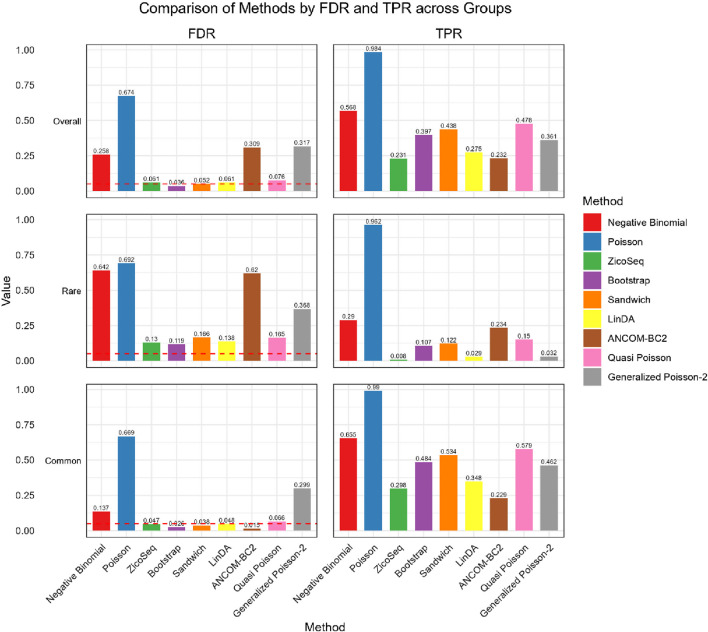
Bar plot of FDR and TPR for ZicoSeq generated simulation with high signal density (30%).

**FIGURE 3:**
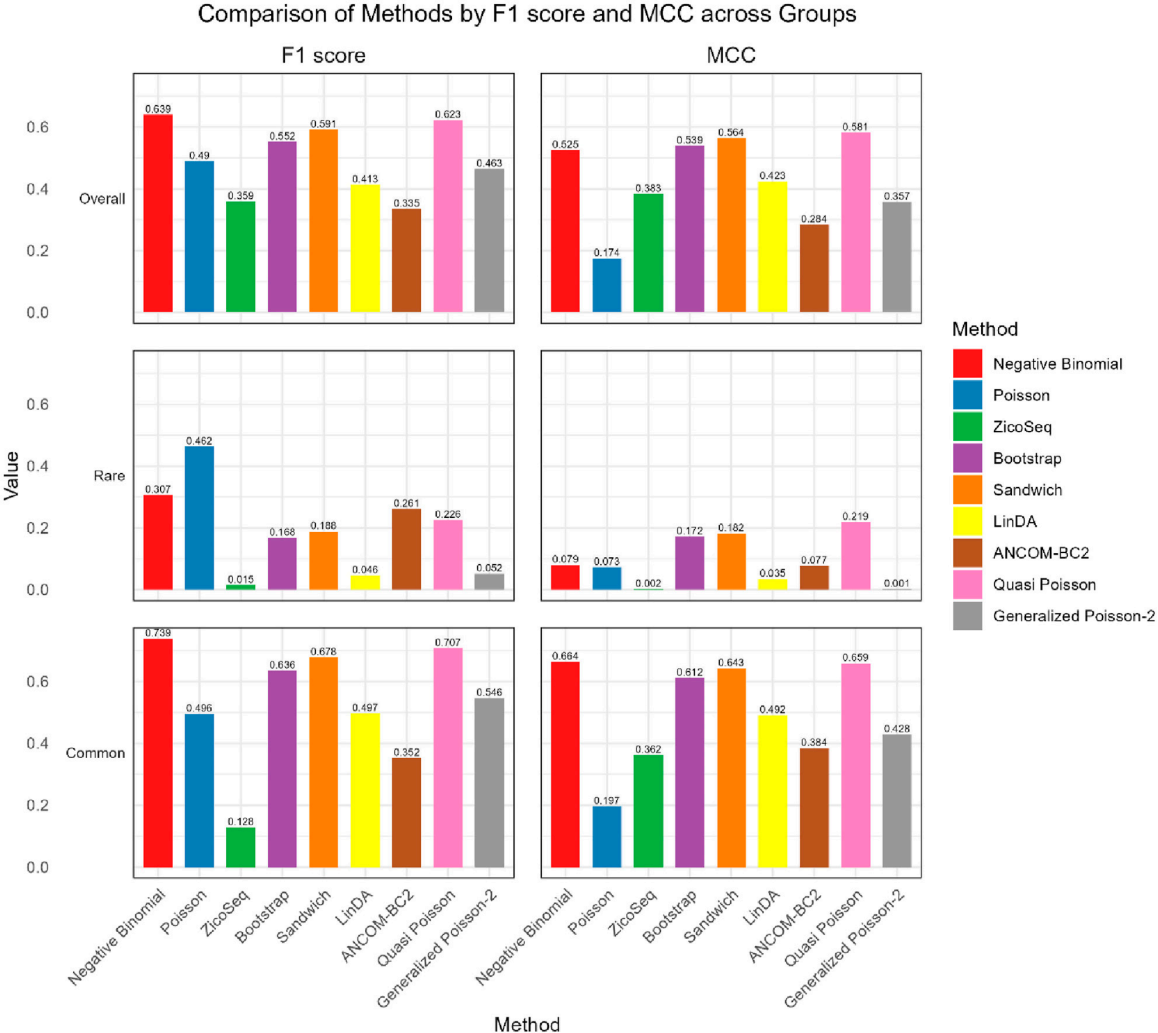
Bar plot of FDR and TPR for ZicoSeq generated simulation with low signal density (10%).

We further compare the performance of the proposed methods in the common and rare groups, to assess their performance under different abundance conditions. The results, shown in Figures 2, 3, indicate that the Negative Binomial regression, Poisson regression, Quasi-Poisson, GP-2 and ANCOM-BC2 models fail to control the false discovery rate around the nominal level. Among these, the ANCOM-BC2 model performs better with the common taxa compared to the rare taxa, suggesting that ANCOM-BC2 requires a higher abundance for effective model fitting. Specifically, we observe convergence issues for the GP-2 model, making it less suitable for applications in microbiome data analysis.

In the simulation setting with high signal density (Figure 2), our proposed bootstrap Poisson model and sandwich Poisson model successfully control the false discovery rate close to the 0.05 nominal level. Additionally, both methods achieve higher statistical power: 0.384 and 0.421 respectively, compared to the power of ZicoSeq (0.241), LinDA (0.278) and ANCOM-BC2 (0.212). Almost all the methods yield false discovery rate more than 10% in the rare taxa (with prevalence from 62.5% to 25%) group. Notably, a severe false discovery inflation (FDR = 0.574) is observed in the ANCOM-BC2 method, in contrast to its good performance of the common taxa group (FDR = 0.031).

We also observe similar patterns in the low signal density groups in Figure 3, indicating the stability of the methods across different signal densities. These results demonstrate that our proposed methods offer higher statistical power in detecting true differential taxa while effectively controlling the false discovery rate.

**FIGURE 4:**
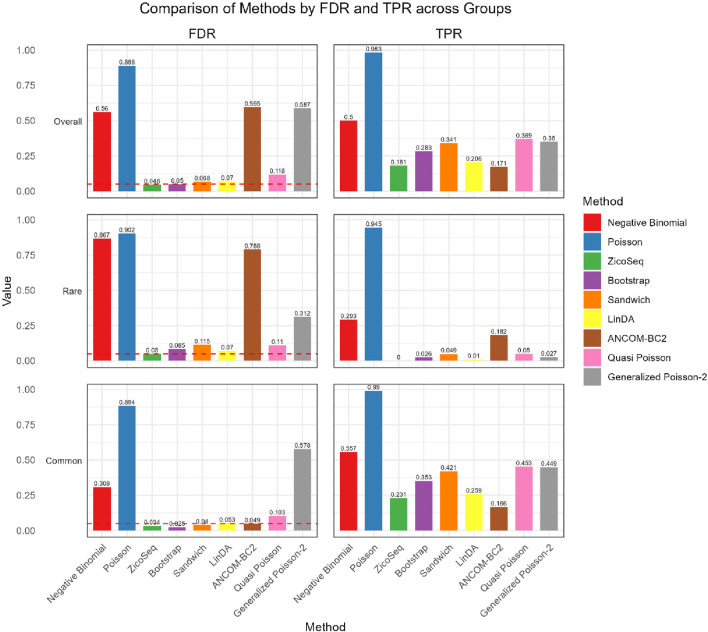
Bar plot of F1 score and MCC for ZicoSeq generated simulation with high signal density (30%).

**FIGURE 5:**
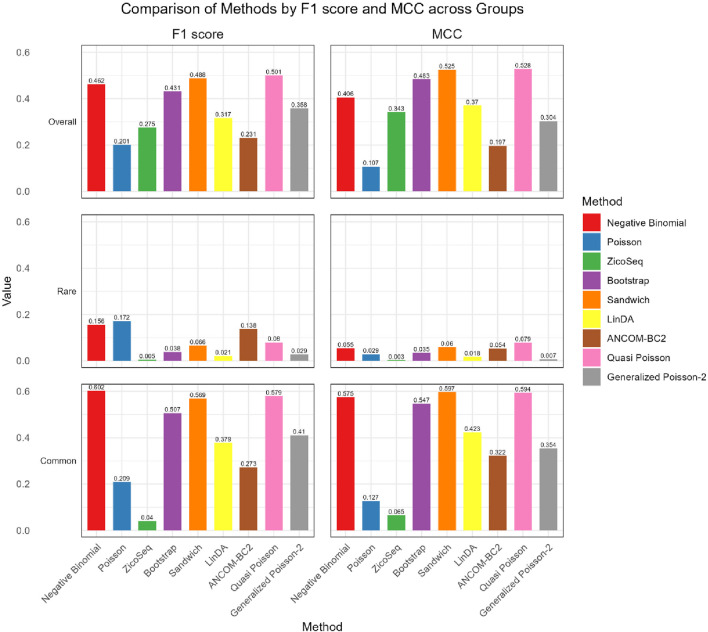
Bar plot of FDR and TPR for ZicoSeq generated simulation with low signal density (10%).

As shown in Figures 4, 5, in both the high and low signal density groups, our proposed Bootstrap Poisson and Sandwich Poisson methods achieve higher F1 scores and MCC than other methods, except for the Negative Binomial regression and Quasi-Poisson (QP) models. However, the NB model and QP model fail to control the false discovery while our proposed methods effectively control the false discovery rate around the nominal level and achieve comparable power. In microbiome studies, particularly in differential analysis, minimizing false positives is crucial due to the large datasets and complex biological signals involved. False positives can lead to incorrect conclusions about the associations between microbial taxa and health outcomes, potentially diverting resources toward investigating spurious findings. This not only misguides scientific understanding but also impacts clinical decision-making if such findings are applied in therapeutic or diagnostic contexts. Therefore, while metrics like the F1 score and MCC are valuable, it is equally important to emphasize the control of false positives to ensure the validity and reliability of the study’s conclusions. Given these considerations, our proposed Bootstrap Poisson and Sandwich Poisson models outperform the alternative models in our simulation study.

## 4 Application

In this section, the proposed Bootstrap Poisson model and Sandwich Poisson model are applied to two real 16S rRNA amplicon sequencing microbiome datasets. The two datasets were collected from human gut and vagina, respectively, which can help us understand the performance of the proposed methods in high- and low-diversity microbial communities. Notably, when fitting the GP-2 model to the two real data in this section, we encounter similar convergence problems to those in the ZicoSeq simulation study. R software generates warnings indicating premature convergence of the regression model, potentially leading to inaccurate and unreliable estimates. Consequently, we exclude it from our real data application section.

### 4.1 Gut samples analysis

In the first study, we apply the proposed methods to the adenomas dataset introduced earlier. Out of the 2147 taxa at the species level collected from 800 fecal samples (266 patients with adenomas and 534 controls), we filter out those with a prevalence of less than 5%, resulting in 102 taxa for testing. The models are fitted to assess the differential abundance of these 102 taxa between the adenomas group and the control group without polyps. The models are adjusted for covariates including gender, batch, and smoking status. Additionally, the log-transformed total count is introduced into the model as an offset to scale the counts of each OTU, equivalent to Total Sum Scaling (TSS). Tests are conducted with the Benjamini–Hochberg (BH) FDR multiple method.

**FIGURE 6:**
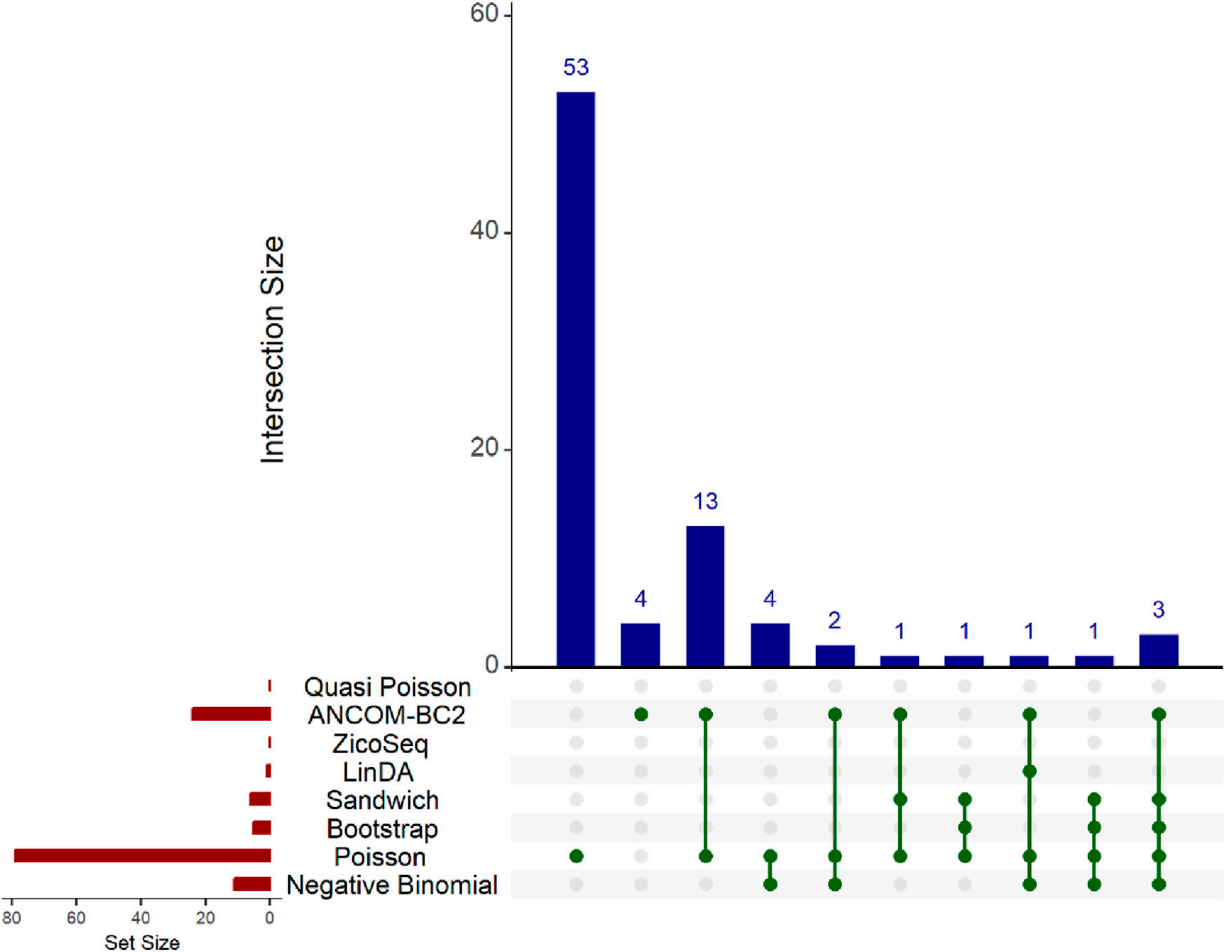
Upset plot for adenomas dataset. The red-colored bar plot at the bottom left shows the number of taxa detected as significant by different methods. The top right blue bar plot displays the size of each intersection, indicating the number of significant taxa shared by the methods corresponding to each intersection. The matrix at the bottom displays the intersections, with each row representing different methods and each column representing a specific intersection. The green dots in the matrix indicate the significant taxa detected by each method. Lines connecting the dots in a column show methods sharing the identified significant taxa.

**FIGURE 7:**
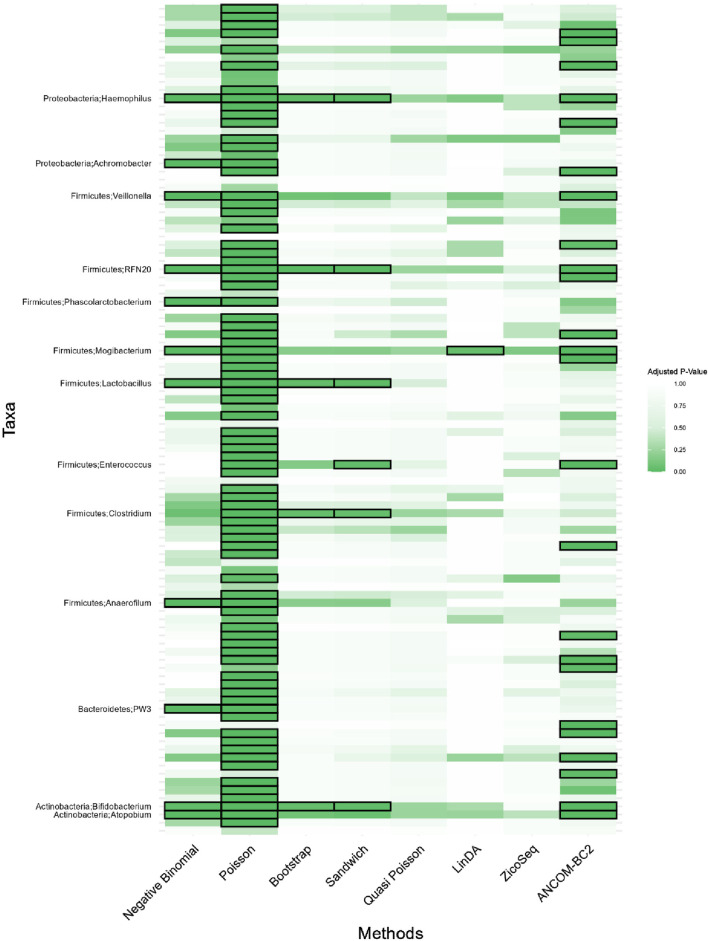
Heatmap for adenomas dataset. Each cell represents the adjusted *p*-value for a specific taxon-method combination, with the color gradient reflecting the magnitude of the *p*-value. Darker shades of green correspond to lower *p*-values, suggesting stronger evidence of differential abundance. The taxa identified by methods other than Poisson or ANCOM-BC2 models are listed on the *y*-axis, while the methods are presented on the *x*-axis. The black boxes highlight the tests that yielded statistically significant results (adjusted *p*-value <0.05).

The results are presented in the Upset plot (Figure 6) and heat map (Figure 7). In the Upset plot, the red colored bar plot at the left bottom panel shows the number of statistically significant taxa detected by different methods. We observe a similar pattern to that in the simulation studies (Figures 2–5). Several taxa are identified as significant by different methods. In the Upset plot, the matrix at the bottom panel shows which sets are involved in each intersection. Each row corresponds to a set, and each column represents a specific intersection. Similar to the simulation study, the Poisson regression model identifies the highest number of significant taxa (79) among all methods, indicating its poor false discovery control under model misspecification and overdispersion. The intersection plot shows that 53 taxa are identified only by the Poisson model, again demonstrating its inconsistency with other models. The ANCOM-BC2 method identifies 24 significant taxa, and the Negative Binomial method identifies 11 significant taxa, all demonstrating poor false discovery control as reflected in Section 3.2 of the simulation studies. The Bootstrap Poisson method identifies 5 significant taxa, all of which are also detected by the Sandwich Poisson method. The Sandwich Poisson method identifies 1 additional significant taxon compared to the Bootstrap method. The LinDA method identifies only 1 significant taxon, while the ZicoSeq and Quasi-Poisson methods fail to identify any significant taxa. In contrast, our proposed methods, Bootstrap Poisson and Sandwich Poisson, demonstrate higher power in detecting differential abundance while maintaining effective false discovery control.

Notably, the genus *Lactobacillus* is identified as significantly associated with adenomatous polyps, precursors to colorectal cancer, by our proposed Bootstrap Poisson and Sandwich Poisson models. *Lactobacillus* is well-known for its probiotic properties, and its presence in the gut has been associated with various health benefits, including the inhibition of colorectal cancer (CRC) progression (Chattopadhyay et al., 2021). Specifically, *Lactobacillus* can inhibit the growth of CRC by reducing inflammation, enhancing intestinal barrier function, and producing short-chain fatty acids like butyrate, which have anti-inflammatory and anti-carcinogenic properties (Profir et al., 2024). This biological support underlines the significance of the *Lactobacillus* detection in our study, providing a strong rationale for its association with colorectal adenomas and further highlighting the robustness of our proposed methods in identifying biologically meaningful taxa.

In contrast, other methods with lower power fail to detect *Lactobacillus*. For example, as shown in Tables 13, 15, the Quasi-Poisson model tends to over-estimate standard errors in over-dispersed distributions (e.g., for *Lactobacillus*), leading to wider confidence intervals and reduced statistical power, which limits its ability to detect significant taxa.

In response to a reviewer’s suggestion, we investigate the biological relevance of the taxa identified as significant by the Poisson model only. Through an extensive literature review, we focus on *Catenibacterium*, *Sutterella*, and *Coprobacillus*—among the top 10 taxa with the lowest *p*-values in the Poisson model, but not flagged by models with better false positive control. Despite their statistical significance, our comprehensive search through PubMed and related scientific databases uncovers no substantial evidence linking these genera to colorectal cancer (CRC). This finding suggests that their detection by the Poisson model may reflect issues with false positive control rather than genuine biological associations.

### 4.2 Vaginal samples analysis

In the second study, we aim to identify associations between vaginal microbiome characteristics during pregnancy and preterm birth (delivery before 37 weeks of gestation). In this study, pregnant patients were enrolled at a single tertiary care institution. Serial mid-vaginal swabs were collected throughout pregnancy during prenatal visits. A nested case-control study was designed to compare samples from patients who delivered at term to those who delivered preterm (Stout et al., 2017). The study included 77 patients, 31% of whom delivered preterm. 149 vaginal swabs were collected: 27 samples during the first trimester, 61 samples from the second trimester, and 61 samples from the third trimester. DNA was extracted using the MO BIO PowerSoil DNA Isolation Kit. Amplicons from the V3V5 regions of the 16S rRNA gene were sequenced using the Roche 454 platform and used in the current analysis.

Given the significant changes in the microbiome community environment during pregnancy (DiGiulio et al., 2015; Berry et al., 2021), we focus on the samples collected from the second trimester by using the first available measurement per individual within this period. This approach ensures consistency in the timing of sample collection, provides a clear baseline measurement of microbiome dynamics during fetal development. We then filter out the OTUs with a prevalence of less than 10% to alleviate zero-inflation, resulting in 31 OTUs and 53 samples in our analysis. We test if the 31 OTU abundance counts are associated with the risk of subsequent preterm birth using the eight models described earlier. The models are adjusted for covariates including race and gestational age, and *p*-values are adjusted using the Benjamini–Hochberg (BH) method for multiple comparisons.

**FIGURE 8:**
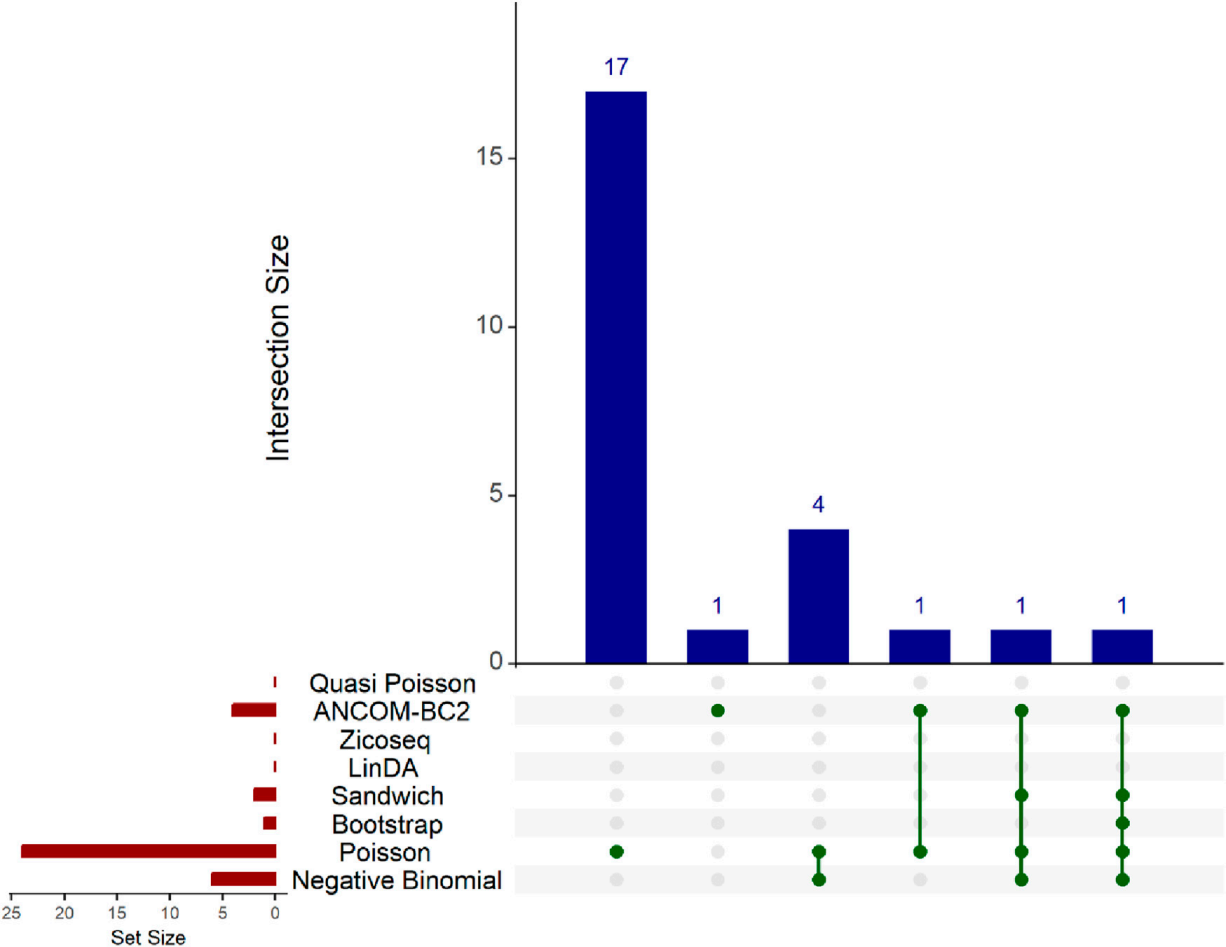
Upset plot for Preterm Birth dataset. The red-colored bar plot at the bottom left shows the number of taxa detected as significant by different methods. The top right blue bar plot displays the size of each intersection, indicating the number of significant taxa shared by the methods corresponding to each intersection. The matrix at the bottom displays the intersections, with each row representing different methods and each column representing a specific intersection. The green dots in the matrix indicate the significant taxa detected by each method. Lines connecting the dots in a column show which methods share the identified significant taxa.

**FIGURE 9:**
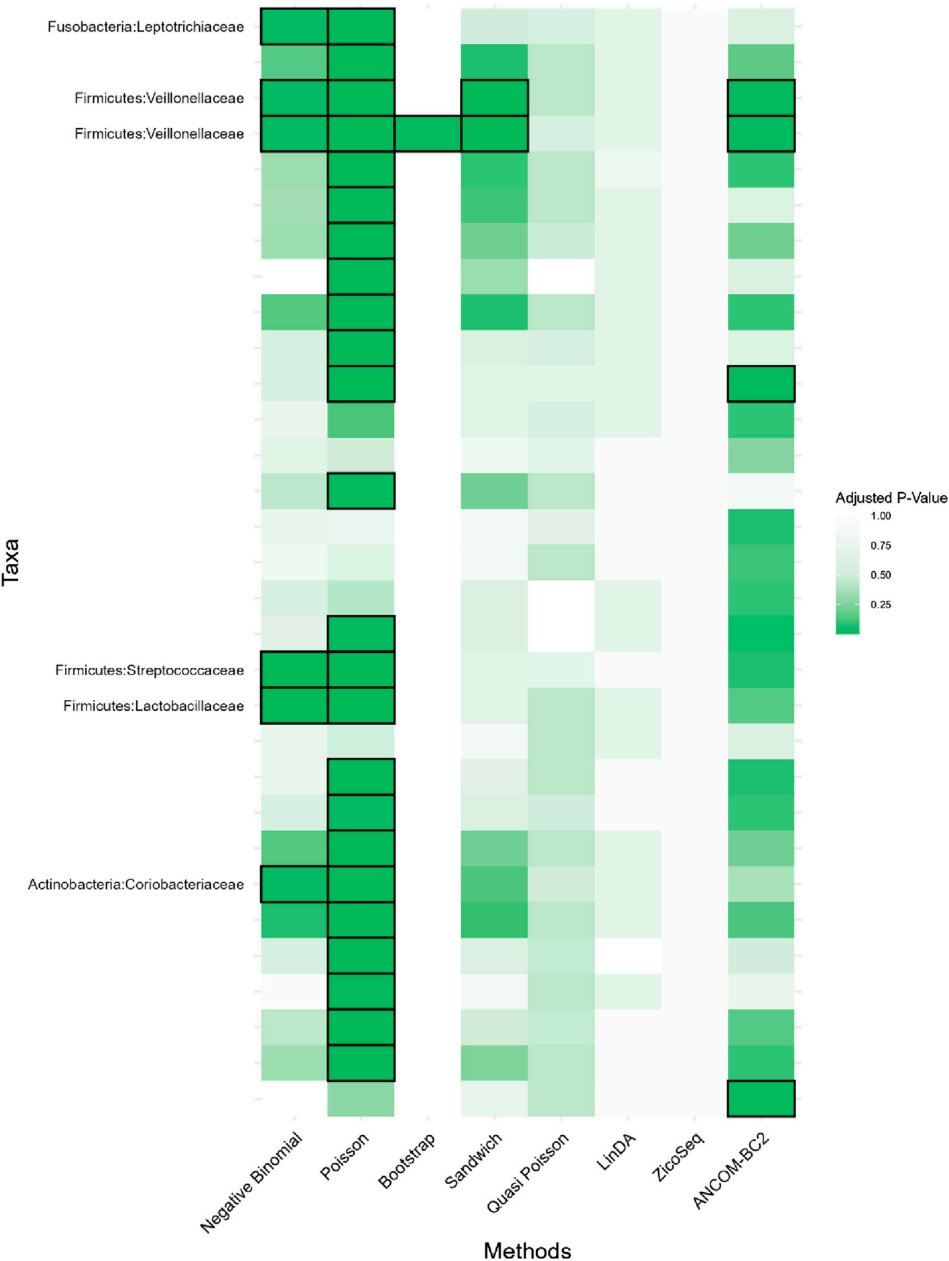
Heatmap for Preterm Birth dataset, each cell represents the adjusted *p*-value for a specific taxon-method combination, with the color gradient reflecting the magnitude of the *p*-value. Darker shades of green correspond to lower *p*-values, suggesting stronger evidence of differential abundance. The taxa identified by methods other than Poisson or ANCOM-BC2 models are listed on the *y*-axis, while the methods are presented on the *x*-axis. The black boxes highlight the tests that yielded statistically significant results (adjusted *p*-value <0.05).

In this real data analysis (Figures 8, 9), Poisson regression identifies the largest number of significant taxa (24), followed by negative binomial regression (6), and then ANCOM-BC2 (4). This reflects the results from the simulation study, where Poisson regression, negative binomial regression, and ANCOM-BC2 fail to control the FDR, leading to an increased number of false positives. The Poisson Bootstrap method identifies 1 significant taxon, which is also significant according to the NB, Poisson, Sandwich and ANCOM-BC2 methods. The Sandwich method additionally identifies 1 significant taxon, also detected by NB, Poisson, ANCOM-BC2. Similar to the performance in the simulation studies, LinDA and ZicoSeq methods fail to find any significant taxa. The QP model again fails to detect any significant taxa, mirroring the results observed in simulation setting (Table 15), where it over-estimates the coefficient’s standard error for over-dispersed distributions and reduces their ability to identify significant associations. Notably, the heatmap plot (Figure 9) shows that the color representing *p*-values for LinDA exhibits a similar trend to those of the Sandwich method. However, there are fewer significant results for LinDA due to its lower power. The results from the vaginal data further validate the effectiveness of our proposed methods, demonstrating their high power in detecting significant differential taxa while effectively controlling false positive findings. In conclusion, the consistent findings across our two different studies affirm the validity and reliability of our methods.

## 5 Discussion

Our study demonstrates that our proposed covariance estimators within Poisson regression effectively mitigates heteroscedasticity in microbiome count data analysis. Extensive simulations show that both methods yield consistent and reliable parameter estimates, even when distributions are mis-specified. Our proposed Bootstrap and Sandwich estimation approaches outperform traditional methods, including negative binomial and Poisson regression, in controlling type I error rates and achieving higher statistical power. When applied to real datasets, such as those involving adenomas and preterm births, the Bootstrap and Sandwich Poisson methods exhibit greater power in detecting significant taxa and offer more accurate control of false discoveries compared to other commonly employed differential abundance analysis techniques. Based on these results, we recommend using these methods for microbiome count data, where the distribution of the count data is unknown and difficult to specify.

While the Bootstrap method is indeed a well-established technique for estimating standard errors and constructing confidence intervals, its application to “partially mis-specified” models, i.e., correct for mean but mis-specified for variance, has not been extensively explored. In our paper, we examine a Poisson model that is correctly specified for the mean ( $\log\mu =X^{T}\beta$ ) but mis-specified for the variance under various scenarios, such as data generated from Gamma, negative binomial, Pareto, and overdispersed Poisson distributions. We demonstrate that, even in these cases, the Bootstrap method remains valid for estimating standard errors. We thus present a concept that has potential for broad generalization: when handling complex data, if the mean structure is correctly specified, parameters can be estimated using a simple model (e.g., Poisson regression) that accurately reflects the mean, even if the variance is mis-specified. Standard errors can then be reliably estimated using the Bootstrap method, which helps to bypass the complexities involved in estimating standard errors in other more intricate models.

The choice between the Bootstrap and Sandwich estimators primarily depends on the computational efficiency required for the analysis. The Bootstrap method is more time-consuming due to its nature of repeatedly fitting Poisson regressions. In contrast, the Sandwich estimator requires significantly less computation time while still providing results that are close to those obtained from the Bootstrap method. Therefore, we recommend using the Bootstrap method when working with relatively small datasets where the additional time required is manageable. For larger datasets, where computational efficiency is a concern, the Sandwich Poisson model is preferable due to its balance between performance and time efficiency. Furthermore, for complex models without readily available sandwich estimates, the Bootstrap method is a practical alternative.

Future research should expand upon our findings to investigate several areas. First, we can combine the two methods with other models, such as Zero-inflated Poisson models, to handle the zero-inflation issue which could further improve accuracy in microbiome data analysis (Xu et al., 2015; Chen and Li, 2016; Hall, 2000). Second, we may explore the application of the proposed methods to longitudinal microbiome samples. A possible solution to this problem is make use of the marginal model (Fitzmaurice et al., 2011). For example, sandwich robust estimators are widely used in Generalized Estimating Equations (GEE) to account for the within-subject correlation and estimate the covariance with working correlation matrix. It is of interest to develop “double decker sandwich” estimates to address within-subject correlation matrix and overdispersion simultaneously.

## Data Availability

The preterm birth vaginal microbiome data presented are deposited in the NCBI Sequence Read Archive (SRA) repository, Bioproject PRJNA294119, accession numbers SRX1524928-SRX1525076. Requests to access the adenomas should be directed to Jun Chen, Chen.Jun2@mayo.edu.
